# Activation Potential of Various Activators for Ferronickel Slag Under Steam Curing: Characterization of Hydration Products and Mechanical Properties

**DOI:** 10.3390/gels12030219

**Published:** 2026-03-06

**Authors:** Yue Li, Baoliang Li, Haohang Yu, Sahi Wail, Binbin Huo, Yongzhen Cheng, Zejun Liu

**Affiliations:** 1 Faculty of Architecture and Civil Engineering, Huaiyin Institute of Technology, Huaian 223001, China; 112205010506@hyit.edu.cn (Y.L.); sr_yuhaohang@126.com (H.Y.); liawisah@gmail.com (S.W.); hylzjgg@hyit.edu.cn (Z.L.); 2 Jiangsu Key Laboratory of Construction Materials, School of Materials Science and Engineering, Southeast University, Nanjing 211189, China; huobinbin@cumt.edu.cn; 3 School of Mines, China University of Mining and Technology, Xuzhou 221116, China

**Keywords:** ferronickel slag, steam curing, alkali-activated slag, MgO, hydrates, microstructure, mechanical properties

## Abstract

This study investigates the activation potential of various activators for ferronickel slag (FNS) and the associated phase evolution. First, the existing forms of MgO in FNS were identified by analyzing its phase composition across different particle sizes. Subsequently, FNS was activated using six types of activators—Ca(OH)_2_, CaO, NaOH, KOH, Na_2_CO_3_, and a Ca(OH)_2_–gypsum composite—under steam curing at 80 °C for 7 days. The setting time, fluidity, hydration products, and mechanical properties of the activated systems were systematically examined. The results show that finer water-cooled FNS particles contain abundant amorphous phases, including amorphous MgO, which can react with Ca-based activators to form hydrotalcite—a reaction not observed with Na- or K-based activators. Compared with Na- or K-based activators, Ca-containing activators, particularly the Ca(OH)_2_–gypsum combination, exhibited superior activation performance. In addition, distinct microstructures were observed: NaOH activation promoted the formation of a yarn ball-like N–A–S–H gel, while KOH activation led to a knotted-fiber-bundle-like K–A–S–H phase, the latter showing potential for enhancing the crack resistance of cement-based materials. These findings provide new insights into the activator-dependent hydration mechanisms of FNS and support its value-added utilization in sustainable construction materials.

## 1. Introduction

Ferronickel slag (FNS) is a solid waste generated during the high-temperature smelting of laterite nickel ore in ferronickel alloy production [1,2]. Based on the production process, FNS can be categorized into blast furnace FNS and electric arc furnace FNS [3], with the latter being the predominant form globally. It is estimated that approximately 14 tons of FNS are discharged per ton of ferronickel alloy produced [4]. With an annual output exceeding 30 million tons in China, FNS has become the fourth-largest smelting industrial waste slag, following iron slag (blast furnace slag), steel slag, and red mud. However, its current utilization rate remains relatively low, at only about 10% [5]. The major applications of FNS include two primary areas: construction materials, where it is used as an aggregate substitute and supplementary cementitious material (SCM) in concrete, and industrial raw materials, for producing magnesium metal, ferroalloys [6], glass fibers [7], glass ceramics [4], and backfill materials [6]. The low utilization rate is partly due to the high magnesium oxide (MgO) content in FNS, which ranges from 2.7% to 31.6% [2,8]. This poses significant challenges to the volume stability of FNS-based composites. Hence, it is crucial to investigate the existing forms of MgO in FNS and its influence on the composition and properties of FNS-derived composites. Moreover, when ground FNS powder is utilized as an SCM in concrete, its reactivity is generally low.

In recent years, the shortage of natural sand has led to a gradual increase in the on-site utilization of FNS sand as a fine aggregate alternative. Consequently, the effects of FNS on concrete durability have attracted growing attention, with a particular focus on the role of MgO. It is known that MgO (in the form of periclase) in cement clinker is classified as dead-burnt. This MgO hydrates slowly to form magnesium hydroxide [Mg(OH)_2_], causing a solid volume increase. This expansion often leads to strength degradation or even concrete cracking [9,10]. For this reason, various standards set a 5% to 8% upper limit for magnesium oxide (MgO) for cement and SCMs [11].

Nevertheless, the reactivity of MgO in FNS is governed by multiple factors, including calcination temperature, cooling rate, particle fineness, and the hydration environment. Based on calcination temperature, MgO is typically categorized into three types: lightly burnt (850–1200 °C), heavily burnt (1500–1800 °C), and dead-burnt (exceeding 1800 °C) [8,12]. As expected, reactivity decreases with increasing calcination temperature. For example, while the hydration of dead-burnt MgO may persist for 6–8 years, lightly burnt MgO can achieve complete hydration within only 3–12 months.

Cooling rate also exerts a critical influence on MgO reactivity in water-cooled slag. A faster cooling rate promotes the formation of more amorphous phases, thereby enhancing the reactivity of slag, and vice versa [13]. For example, granulated blast furnace slag (GBFS) undergoes rapid cooling, and its MgO can participate in hydration reactions under high-alkalinity conditions, forming hydrates such as hydrotalcite [14]. In contrast, slow cooling facilitates the crystallization of MgO into stable phases such as forsterite, which exhibits negligible hydration activity at room temperature [8].

Mechanical activation is a widely adopted technique for producing micron- and submicron-scale particles via grinding. This method enhances the reactivity of MgO-containing phases by introducing lattice defects in the crystal structure and increasing the particles’ specific surface area [15]. In addition, the hydration environment is another key factor affecting MgO reactivity. At ambient temperature and pressure, the hydration of MgO in cement paste is significantly delayed [16]. In contrast, steam curing at 80 °C can effectively accelerate the hydration of lightly burnt MgO, allowing for its complete hydration within 30 days [12]. More remarkably, autoclave curing (216 °C, 2 MPa steam pressure, 3 h) can raise the MgO hydration degree to nearly 72% within a short period. For this reason, standards such as those from the American Society for Testing and Materials (ASTM) have adopted autoclave curing as a method to evaluate the soundness of MgO-containing binders [17].

Rahman et al. [8] reported that MgO in water-cooled FNS exists as forsterite rather than periclase. Similarly, Zhai et al. [5] confirmed that MgO in FNS is predominantly present in crystalline phases such as clinoenstatite and forsterite, and these phases do not induce hydration expansion. However, it remains unclear whether other forms of MgO are present in FNS and, if so, how they affect the performance of FNS-based composites.

Low reactivity represents another key constraint on the utilization of FNS in concrete. Due to its inherent characteristics, FNS is generally not classified as a hydraulic material. To enhance the reactivity of FNS, the technical measures currently employed are summarized as follows: (1) Mechanical activation (grinding): Wet grinding has been demonstrated to be highly effective in boosting FNS reactivity; specifically, a 20–60 min wet-grinding process can increase the 28-day activity index of FNS by 18.0% to 24.7% [18]. (2) Steam curing: The authors’ findings indicated that early-age steam curing at 80 °C for 7 h increased the 28-day compressive strength activity index of cement mortar incorporating 20% FNS powder, with the value rising from 77.5% to 85.7% [2]. (3) Alkali activation: The presence of SiO_2_ and Al_2_O_3_ in the amorphous phase of FNS endows it with potential for the preparation of alkali-activated materials (AAMs). Cao et al. [19] evaluated the effects of FNS on GBFS activated by alkalis (NaOH and water glass) and found that FNS incorporation mitigates the drying shrinkage of AAMs without compromising their mechanical properties. Nevertheless, the setting time of alkali-activated FNS exceeds 24 h owing to the low amorphous content of FNS, which suggests that the sole alkali activation of FNS is not a viable approach.

However, it has not been demonstrated whether FNS can be effectively activated by combining steam curing with alkali treatment. Additionally, the effectiveness of different alkali types for activating FNS remains unknown, despite their wide use in AAM synthesis.

A key application of FNS is as an SCM in concrete. Notably, concrete contains several components—such as Ca(OH)_2_, CaSO_4_∙2H_2_O, Na ions, and K ions—that are also common alkali activators in AAM systems. Given this compositional overlap, it remains unclear how effectively these concrete-derived components can activate FNS.

Therefore, to explore the existing forms of MgO in FNS and enhance its reactivity, this study first characterized the phase and chemical compositions of FNS samples with different particle sizes. Subsequently, the hydration products and mechanical properties of alkali-activated FNS under steam curing conditions were systematically investigated, with particular emphasis on the effects of alkaline activators. Specifically, six types of activators were selected for this work, namely calcium hydroxide (Ca(OH)_2_), calcium oxide (CaO), sodium hydroxide (NaOH), potassium hydroxide (KOH), sodium carbonate (Na_2_CO_3_), and a composite activator consisting of Ca(OH)_2_ and gypsum.

The results confirm that the existing forms of MgO in FNS are governed by both its particle size and cooling rate. Moreover, the chemical properties of the activators exert a decisive influence on the phase composition and strength development of alkali-activated FNS. Unlike alkali-activated GBFS systems, the formation of hydrotalcite in alkali-activated FNS exhibits a stronger dependence on calcium-containing activators. Moreover, under steam curing conditions, activators containing calcium hydroxide demonstrate particularly pronounced activation effects on FNS. These findings provide a theoretical basis for the efficient utilization of FNS; additionally, they deepen the understanding of activator effects on the hydration mechanisms of both alkali-activated FNS and FNS incorporated in concrete.

## 2. Results and Discussion

### 2.1. Results of Material Characterization

The chemical composition of ferronickel slag (FNS), determined via X-ray fluorescence (XRF) spectroscopy, is presented in Table 1; the results demonstrated that, in comparison with conventional SCM like ground granulated blast furnace slag (GBFS), the FNS sample showed elevated concentrations of SiO_2_, MgO, and Fe_2_O_3_, alongside reduced contents of CaO and Al_2_O_3_. It is noteworthy that the MgO and Fe_2_O_3_ contents in this FNS reached 15.94% and 13.24%, respectively, which are substantially higher than those typically found in ordinary SCMs.

**Figure 1:**
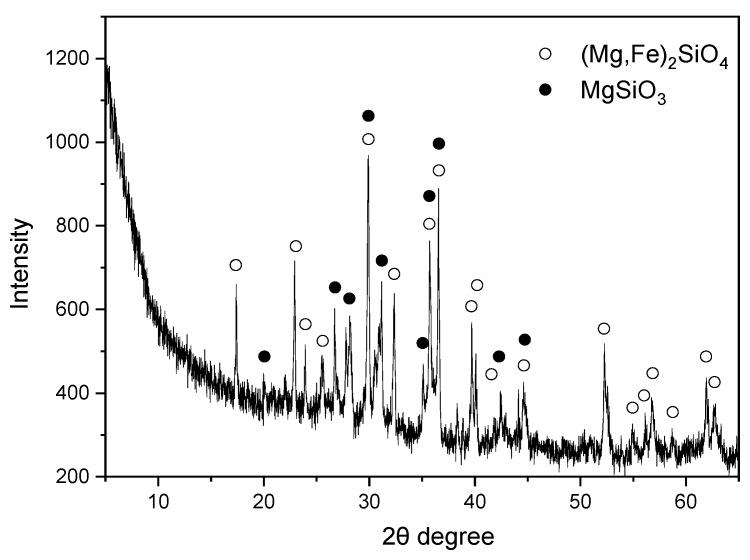
X-ray diffraction pattern of FNS powder.

X-ray diffraction (XRD) analysis (Figure 1) reveals that, in addition to crystalline forsterite ((Mg, Fe)_2_SiO_4_) and enstatite (MgSiO_3_), the FNS contained a small amount of amorphous phase. Their relative contents determined via the XRD/Rietveld method were 49.69% forsterite, 20.19% enstatite, and 30.12% amorphous phase. Furthermore, it is noteworthy that no periclase (free MgO) was detected.

**Figure 2:**
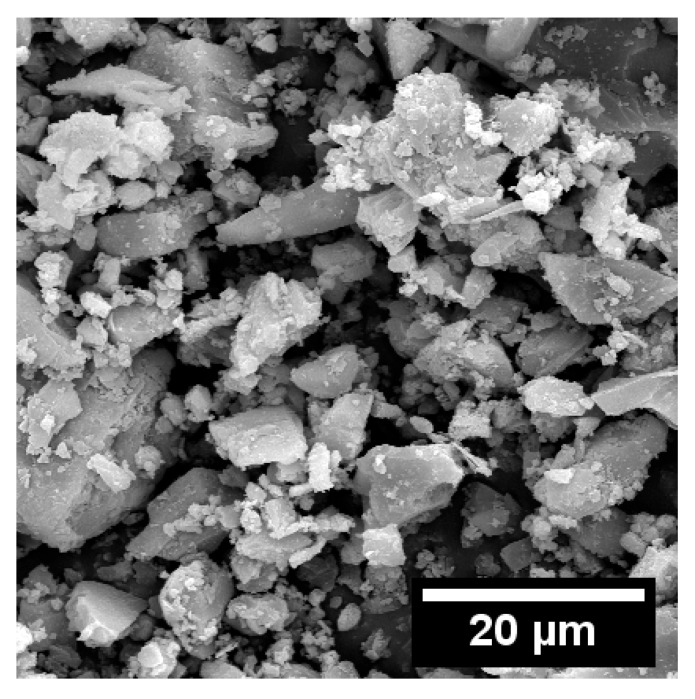
The morphology of FNS powder under SEM.

Scanning electron microscopy (SEM) was used to characterize the morphology of the FNS powder, with the corresponding micrographs shown in Figure 2. FNS particles exhibited a gravel-like morphology with irregular shapes and no distinct edges, and their particle size was predominantly in the range of 10–20 μm.

**Figure 3:**
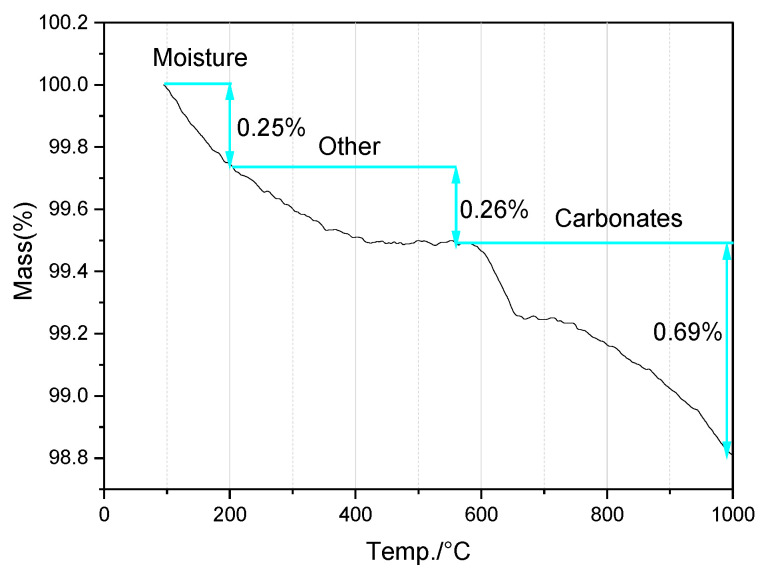
The TG curve of FNS powder.

The thermogravimetric (TG) curve in Figure 3 suggests that the loss on ignition (LOI) of the FNS was approximately 1.2%; this value is related not only to physically adsorbed water (moisture) and the decomposition of carbonate phases, but also to the decomposition of some hydroxides, sulfides, and organic matter.

**Figure 4:**
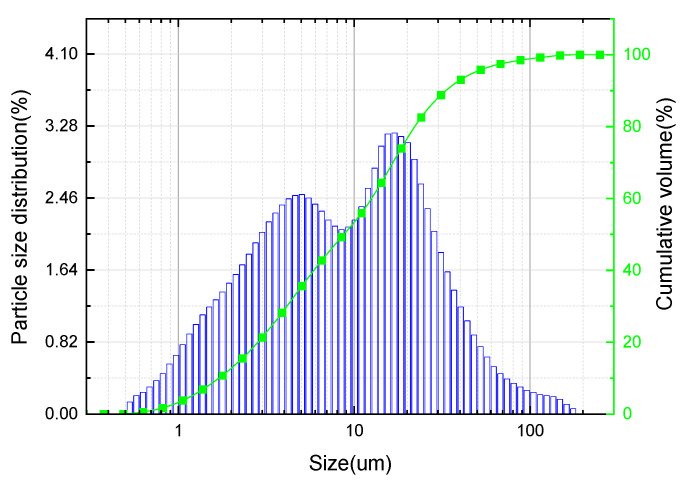
Particle size distribution of FNS powder.

Laser particle size analysis shows that the particle size of FNS powder was primarily in the range of 3 μm to 30 μm, with an average particle size of 8.6 μm (Figure 4); its most probable particle size was 18.86 μm, which was consistent with the SEM results.

### 2.2. The Occurrence Forms of MgO in FNS

**Figure 5:**
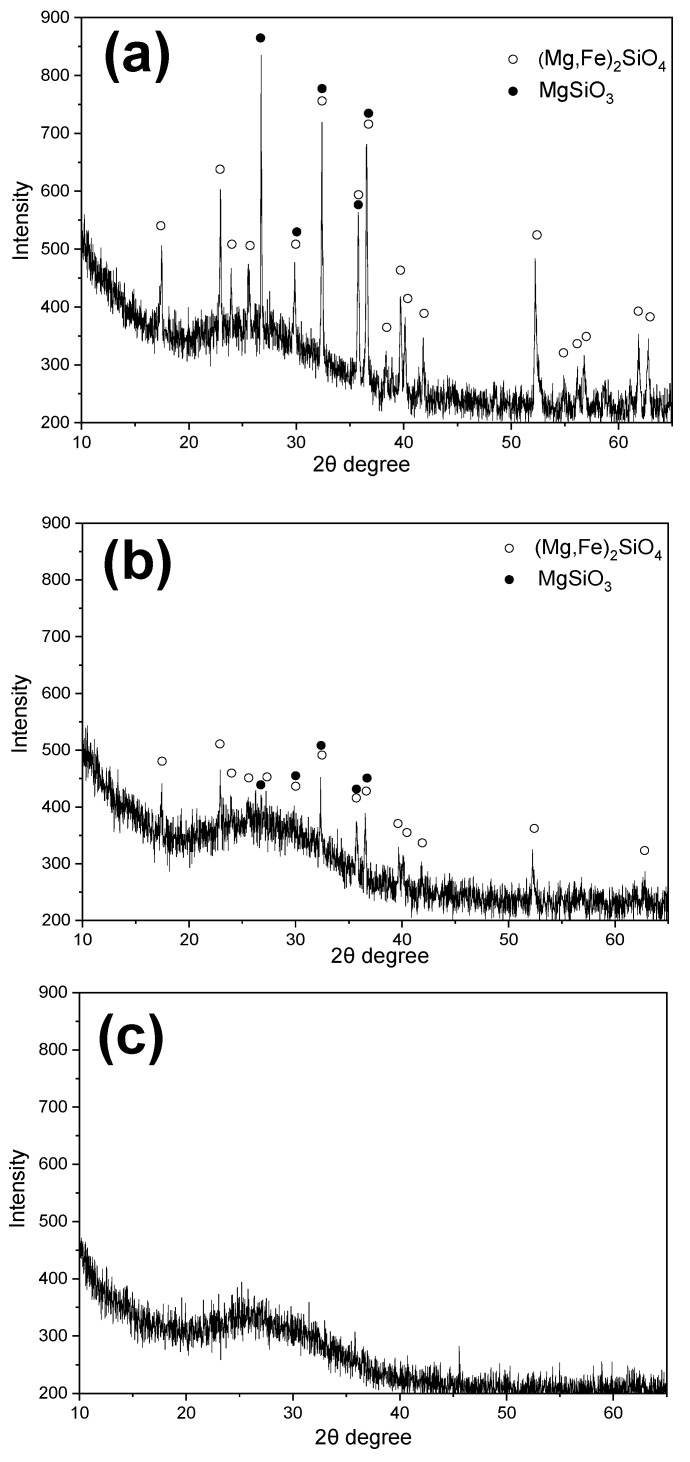
XRD results of FNS sand with various particle sizes: (**a**) larger than 4.75 mm, (**b**) 0.15 mm~4.75 mm, (**c**) smaller than 0.15 mm.

This study was inspired by the high reactivity of MgO in water-quenched GBFS and aimed to investigate the occurrence forms of MgO in FNS. Water-cooled FNS particles of different sizes (from the same source) were selected and ground to a particle size of less than 0.08 mm. Subsequently, XRD and XRF analyses were conducted to determine their phase and composition, the results of which are shown in Figure 5 and Table 2. It is interesting that more crystalline phases appeared in FNS with particle sizes greater than 4.75 mm and particle sizes between 0.15 mm and 4.75 mm, while almost amorphous phases appeared in FNS with particle sizes less than 0.15 mm, and no obvious crystal mineral diffraction peak was identified in Figure 5c. This is undoubtedly related to the fact that the cooling rate of small particles is faster than that of large particles during water cooling, indicating that the cooling rate or particle size is also an important factor to determine the existence form of the phase in water-cooled FNS. The faster the cooling rate or the smaller the particle size, the more the amorphous phase generated; therefore, the higher the reactivity of FNS and vice versa. Consequently, FNS can be classified for use; that is, FNS with large particle size can be used as aggregate, and FNS with small particle size can be ground to be used as SCM for concrete.

In addition, it can be seen from Table 2 that the chemical compositions of FNS with different particle sizes showed no obvious differences, and all contained approximately 20% MgO. Combining the results of Figure 5 and Table 2, it can be inferred that MgO in FNS existed mainly in two forms, namely the mineral crystalline phase (in (Mg, Fe)_2_SiO_4_ and MgSiO_3_) and the amorphous phase. Crystalline MgO was relatively stable and difficult to react at room temperature [8], whereas amorphous MgO might participate in the formation of hydration products.

### 2.3. Setting Time and Fluidity

#### 2.3.1. Setting Time

In order to compare the reactivity of FNS with various alkali activators and the workability of alkali-activated FNS paste, the setting time and fluidity of alkali-activated FNS paste were tested, and the results were stated in Table 3. In Table 3, the abbreviations FC, FCO, FCS, FN, FNC, and FK correspond to FNS activated by Ca(OH)_2_, CaO, Ca(OH)_2_ + gypsum, NaOH, Na_2_CO_3_, and KOH, respectively.

It is unsurprising that the hydration and hardening of alkali-activated FNS required a significantly long time at room temperature, with the exception of the sample containing CaO as the activator. The dissolution of CaO releases substantial heat (64.45 kJ/mol), which is responsible for the short setting time of the FCO paste [20]. Although Ca(OH)_2_ (abbreviated as CH) is chemically similar to CaO, the setting time of the sample based on CH was much longer. This can be attributed to several factors: (1) The dissolution of CH liberates less heat (17.8 kJ/mol) than that of CaO [20]; (2) Under the same mass condition, CH accounts for a greater proportion in FCO (1 g of CaO can ultimately yield 1.32 g of CH in the presence of sufficient water); (3) The transformation from CaO to CH consumes free water, thereby reducing the water-binder ratio of the mixture.

The coupling effects of both CH and gypsum can accelerate the hardening of FNS paste; thus, the setting time of FCS was shorter than that of FNS activated by CH alone. The reactive Al derived from FNS can react with CH and gypsum in aqueous solution to form ettringite (AFt) or monosulfoaluminate hydrate (AFm), which may be responsible for the shorter setting time of FCS [21].

As a strong base, NaOH resulted in a shorter setting time for FNS compared to the other four activators, except for CaO. Although KOH has higher alkalinity and a greater tendency to combine with aluminosilicate anions than NaOH, the setting time of the KOH-activated paste was longer than that of the NaOH-activated paste. This finding aligns well with the literature [22]. The main possible reasons are as follows: (1) The molecular weight of KOH (~56) is higher than that of NaOH (~40). Therefore, at the same mass, NaOH provides a greater molar amount, which releases more Na^+^ and OH^−^ ions into the aqueous solution. (2) Mixtures activated by NaOH release more hydration heat at an early age than those activated by KOH under the same conditions [23].

Compared to other samples, the sodium carbonate-based paste exhibited the longest setting time; given that sodium carbonate is inherently a weak base, this result is not coincidental. As a weak alkali, Na_2_CO_3_ provides a considerably lower OH^−^ concentration in aqueous solution than NaOH or KOH solutions at the same concentration. This lower OH^−^ concentration leads to a slower depolymerization rate of FNS, thereby reducing the release of silicate and aluminate ions. Consequently, the formation of hydration products and the establishment of the network structure are delayed, resulting in a significantly prolonged setting time. Secondly, the carbonate ions (CO_3_^2−^) introduced by Na_2_CO_3_ may combine with Ca^2+^ ions released from the FNS hydration products to form CaCO_3_ precipitates. On one hand, these precipitates may cover the surface of FNS particles, hindering further depolymerization. On the other hand, they may compete with hydration products for available calcium ions, thereby affecting the formation rate and microstructure of C–S–H gel [24]. Therefore, under the combined influence of these two factors, the Na_2_CO_3_-activated system exhibits the longest setting time.

#### 2.3.2. Fluidity

Based on their chemical nature, the six activators used in this study can be classified into Ca-based activators (i.e., CH, CaO, and the CH–gypsum composite system) and Na(K)-based activators (NaOH, Na_2_CO_3_, and KOH). As shown in Table 3, the mixtures activated by Na(K)-based activators exhibited significantly higher fluidity than those activated by Ca-based ones. This difference is likely attributable to the lower solubility of Ca-based activators [25,26]. Among the Na(K)-based activators, no significant difference in fluidity was observed. Although literature suggests that KOH-activated mixes generally have lower plastic viscosity and yield stress than NaOH-activated ones, leading to better fluidity [22], all Na(K)-activated pastes in this study showed excessively high fluidity, making it difficult to distinguish between them. For the Ca-based activators, the paste containing only CH (FC) demonstrated higher fluidity than those with CaO (FCO) or the CH-gypsum composite (FCS). This can be explained by the fact that CaO hydration consumes a considerable amount of free water, and gypsum can participate in forming AFt or AFm phases, which incorporate substantial water of crystallization in the presence of reactive Al and CH.

### 2.4. Hydrates and Microstructure Determined by XRD, TG/DTG and SEM-EDS

#### 2.4.1. FNS Activated by Ca(OH)_2_

**Figure 6:**
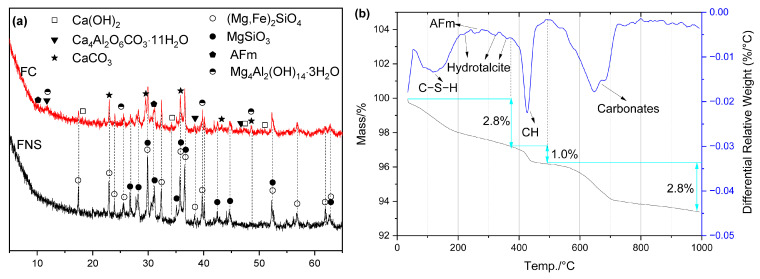
Hydration products of FC: (**a**) XRD curves; (**b**) TG/DTG curves.

**Figure 7:**
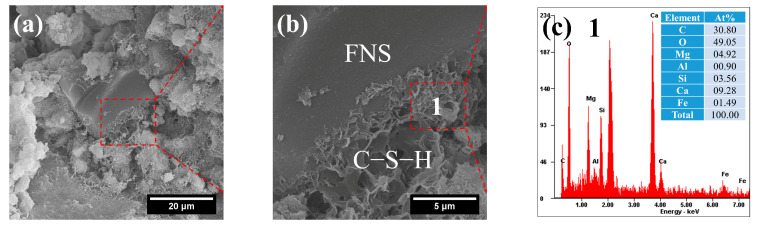
SEM-EDS images of FC: (**a**) unreacted FNS; (**b**) reticular C–S–H gels; (**c**) composition of C–S–H gels.

**Figure 8:**
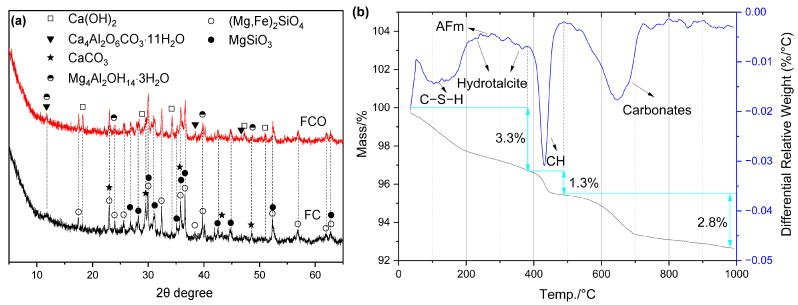
Hydration products of FCO: (**a**) XRD curves; (**b**) TG/DTG curves.

**Figure 9:**
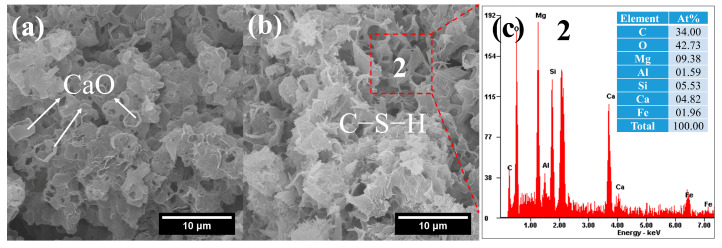
SEM-EDS images of FCO: (**a**) reacted CaO; (**b**) compact C–S–H gels; (**c**) composition of C–S–H gels.

**Figure 10:**
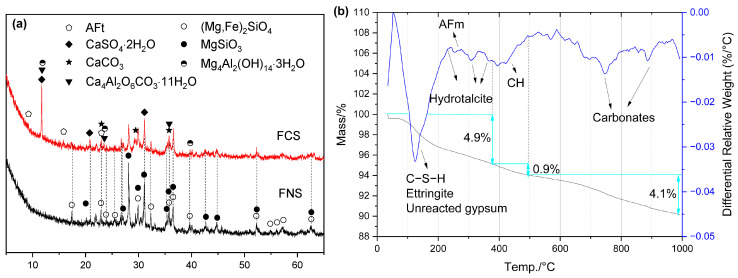
Hydration products of FCS: (**a**) XRD curves; (**b**) TG/DTG curves.

**Figure 11:**
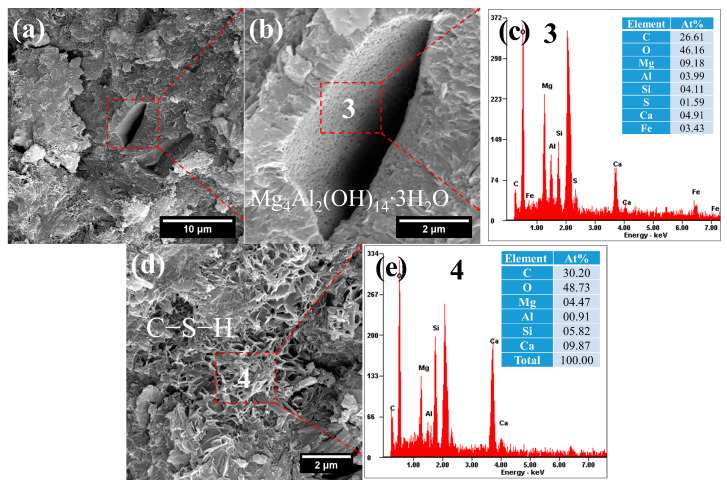
SEM-EDS images of FCS: (**a**) hydrotalcite; (**b**) hydrotalcite; (**c**) composition of hydrotalcite; (**d**) loose C–S–H gels; (**e**) composition of C–S–H gels.

**Figure 12:**
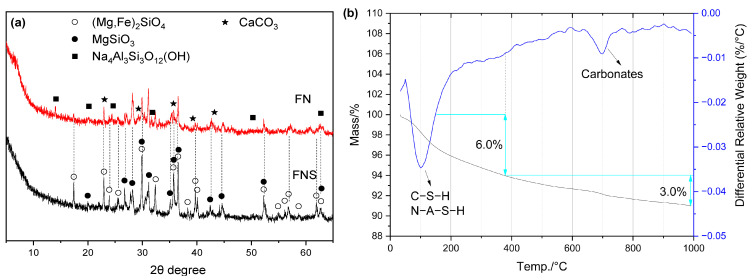
Hydration products of FN: (**a**) XRD curves; (**b**) TG/DTG curves.

**Figure 13:**
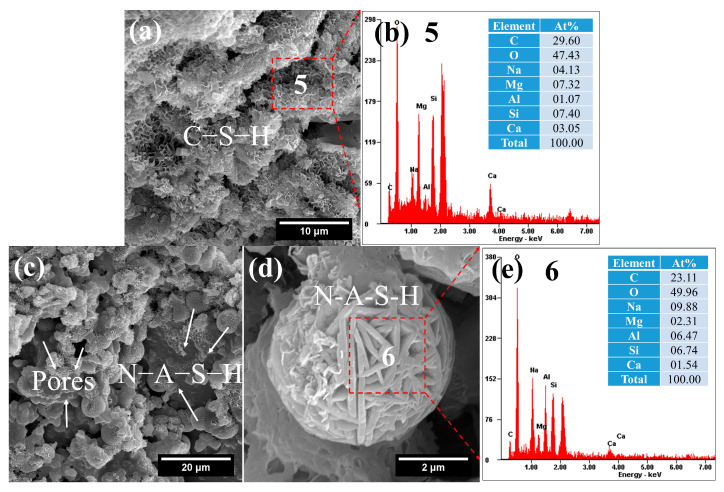
SEM-EDS images of FN: (**a**) reticulated C–S–H gels; (**b**) composition of C–S–H gels; (**c**) pores and yarn ball-like N–A–S–H; (**d**) yarn ball-like N–A–S–H; (**e**) composition of N–A–S–H.

**Figure 14:**
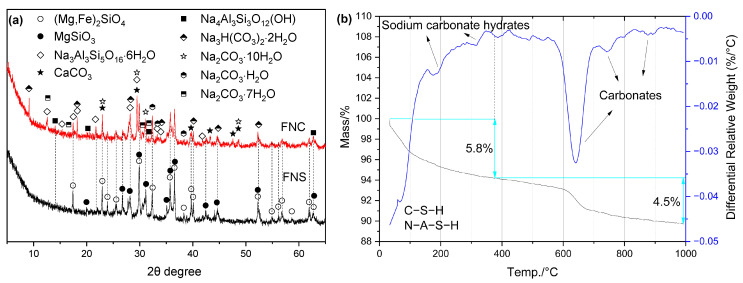
Hydration products of FNC: (**a**) XRD curves; (**b**) TG/DTG curves.

**Figure 15:**
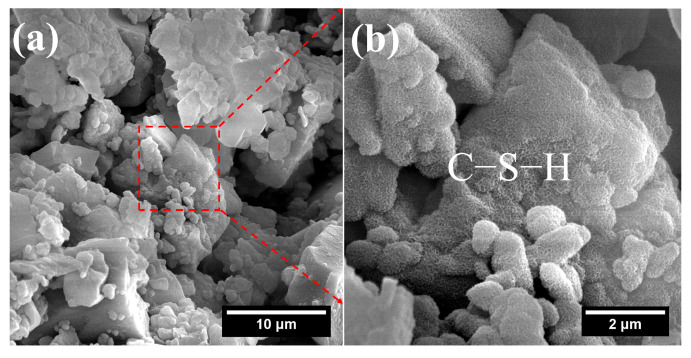
SEM-EDS images of FNC: (**a**) microstructure of the hardened paste; (**b**) C–S–H.

**Figure 16:**
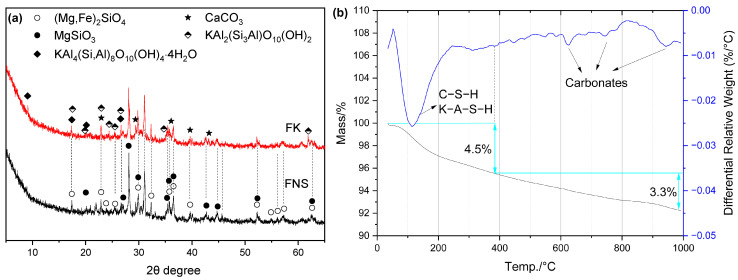
Hydration products of FK: (**a**) XRD curves; (**b**) TG/DTG curves.

**Figure 17:**
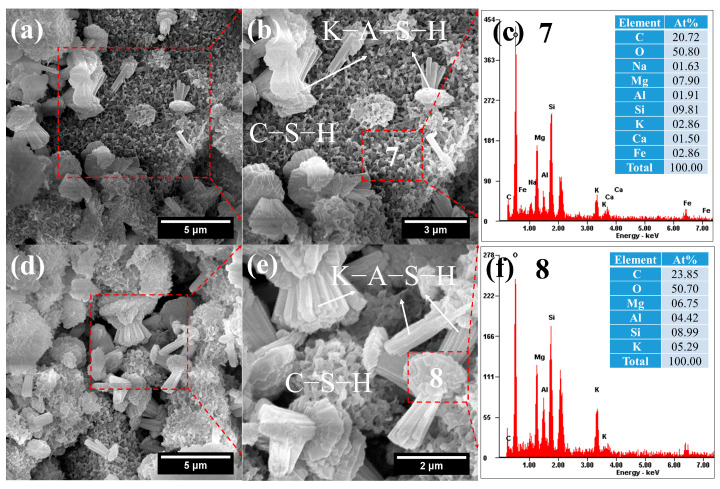
SEM-EDS images of FK: (**a**) honeycomb-like C–S–H gels; (**b**) honeycomb-like C–S–H gels; (**c**) composition of C–S–H; (**d**) knotted-fiber-bundle-like K–A–S–H; (**e**) knotted-fiber-bundle-like K–A–S–H; (**f**) composition of K–A–S–H.

To investigate the effects of various activators on hydration products, the hydration phases in alkali-activated FNS were characterized using XRD, TG/DTG, and SEM-EDS, as shown in Figure 6, Figure 7, Figure 8, Figure 9, Figure 10, Figure 11, Figure 12, Figure 13, Figure 14, Figure 15, Figure 16 and Figure 17. Figure 6 shows that C–S–H gels, monocarbonate (Ca_4_Al_2_O_6_CO_3_·11H_2_O), AFm, hydrotalcite (Mg_4_Al_2_(OH)_14_·3H_2_O), and CaCO_3_ were identified in the FC sample, along with residual forsterite and enstatite from the FNS. Notably, strätlingite (C_2_ASH_8_), a common hydration product in lime-activated cementitious systems, was not observed. This absence may be attributed to insufficient aluminum content in the FNS [27]. It is also worth noting that the formation of monocarbonate is primarily due to the presence of carbonates in the FNS (Figure 3).

As shown in Figure 6, the TG-DTG results reveal that the primary hydration products of FC were C–S–H, AFm, hydrotalcite, and calcium carbonate, which is in agreement with the XRD findings. Based on the TG-DTG data, the total mass loss and the differential mass losses within key temperature ranges (<390 °C, 390–490 °C, >490 °C) for various alkali-activated FNS hardened pastes were calculated, as summarized in Table 4. The mass loss at temperatures below 390 °C is primarily due to the dehydration and decomposition of hydration products, including C–S–H, AFm, and hydrotalcite. This mass loss correlates with the non-evaporable water content, thereby indicating the hydration degree of the alkali-activated FNS system. The mass loss between 390 and 490 °C is mainly attributed to the dehydroxylation of portlandite (CH), while the mass loss above 490 °C chiefly results from the decomposition of calcium carbonate (CaCO_3_). For reference, the typical thermal decomposition temperature ranges of the relevant phases investigated in this study were listed in Table 5.

Figure 6b and Table 4 show that, in addition to unreacted CH, carbonates constituted a significant proportion of the hydration products. These carbonates have minimal influence on mechanical properties. CH is highly susceptible to CO_2_ and is one of the most readily carbonated hydrates in cement-based materials [39]. During carbonation, a dense CaCO_3_ layer forms on the mixture surface, densifying the paste structure. While this can sometimes contribute to strength development, it simultaneously reduces the overall alkalinity of the paste. Furthermore, metastable phases such as monocarbonate will ultimately transform into more stable forms of CaCO_3_, such as calcite or vaterite [40,41]. Carbonation of C–S–H also generates various CaCO_3_ polymorphs [39]. It should also be noted that a small amount of carbonate was originally present in the FNS (Figure 3). Therefore, the carbonates shown in Figure 6b are attributable not only to carbonation but also to unreacted FNS residues. The minor amount of primary carbonate present in the FNS (Figure 3) is generally regarded as a relatively inert phase. During the early stages of alkali-activated hydration, it does not directly participate in the principal chemical reactions but instead acts as a micro-aggregate or filler within the matrix, thereby influencing the compactness of the paste [42].

Notably, during sample preparation and transportation, the alkali-activated FNS is susceptible to carbonation upon exposure to CO_2_, leading to the formation of secondary carbonates. The impact of these secondary carbonates on hydration products and macroscopic properties is more intricate and is governed by the timing and extent of carbonation. The carbonation process generates substantial amounts of CaCO_3_, typically crystallizing as calcite or aragonite. To a certain extent, these CaCO_3_ crystals can fill pores and enhance the compactness of the matrix; however, excessive carbonation may lead to the decomposition of hydration products such as C–S–H, thereby compromising the cementitious capacity of the material.

Although the content of SO_3_ in FNS was small (Table 1), it seems that CO_3_^2−^ from carbonates and the additional OH^−^ in the FNS-CH system can participate in the formation of AFm instead of SO_4_^2−^ [43,44]. It is gratifying that the appearance of hydrotalcite proves MgO in the amorphous phase of FNS can take part in the reaction, which is in good agreement with the above analysis.

At room temperature, the pH of a saturated Ca(OH)_2_ solution is only about 12.5 [45], which is relatively low. Long-term steam curing, however, can compensate for this low alkalinity. Under the coupled effects of elevated temperature and an alkaline environment, reactive SiO_2_ and Al_2_O_3_ in the amorphous phase of FNS dissolve and participate in forming hydration products such as C–S–H gels and AFm phases. This process ultimately provides strength to FNS activated by CH.

Figure 7 displays the morphology and composition of C–S–H gels in FC. Reticular C–S–H gels near the surface of unreacted FNS particles were observed, which is similar to that of pure cement [46]. EDS results show that the Ca/Si ratio of C–S–H is around 2.6 (Figure 7c). The larger Ca/Si ratio of the C–S–H may be attributed to its intergrowth with CH in the mixture. 

Although the appearance of Mg in the composition of C–S–H was also identified, it is difficult to prove that Mg is involved in the formation of C–S–H due to the large difference between M–S–H and C–S–H structure [47]. According to the literature [47], the ionic radius of Ca^2+^ in the octahedral layer of C–S–H is 0.99 Å, whereas that of Mg^2+^ is only 0.65 Å. This substantial size difference precludes the substitution of Mg^2+^ for Ca^2+^ in the octahedral sites of the C–S–H structure. Consequently, the Mg signals detected in the EDS analysis are more likely attributable to the intergrowth of hydrotalcite with C–S–H phases.In addition, the limited amorphous content in FNS resulted in fewer products in FC, which was consistent with the TG results, and also indicated that the strength of FC was low.

#### 2.4.2. FNS Activated by CaO

Since CaO can react with water to produce Ca(OH)_2_, the types of hydrates in FCO were similar to those in FC, as shown in Figure 8. In contrast, FCO exhibited a higher non-evaporable water content and a greater amount of residual CH compared to FC, as observed in Figure 8b and Table 4.

The morphology and composition of hydration products in FCO are shown in Figure 9. Hollow and incomplete crystal shells, likely representing incompletely reacted CaO, can be observed near the surface of C–S–H (Figure 9a). In addition, the C–S–H in FCO exhibited a more compact structure and a lower Ca/Si ratio compared to that in FC, indicating the potential for relatively higher strength in FCO. Typically, a lower Ca/Si ratio in C–S–H correlates with a higher degree of silicate polymerization and a denser gel structure, leading to improved mechanical performance in cement-based materials [48,49].

CaO plays an almost identical role to CH in the activation of FNS. However, each gram of CaO can yield up to 1.32 g of Ca(OH)_2_ when fully hydrated. This hydration reaction not only releases additional heat [20], which accelerates the dissolution of amorphous phases in FNS and thereby promotes the overall alkali-activation process, but also consumes free water in the mixture. The reduction in free water enhances the compactness of the resulting matrix.

#### 2.4.3. FNS Activated by the Combination of Ca(OH)_2_ and Gypsum

Under the combined activation of gypsum and CH, the formation of both ettringite and AFm phases was further confirmed, as identified in Figure 10. Although ettringite is known to decompose at elevated temperatures, its formation was still observed after 7 days of steam curing at 80 °C, which can be attributed to the presence of excess gypsum (Figure 10a). Furthermore, FCS exhibited a higher non-evaporable water content than FC and FCO (Table 4). This increase is primarily attributed to the formation of additional ettringite and a higher content of C–S–H gel. The lower content of unreacted CH in FCS corroborates this enhanced degree of reaction.

SEM-EDS analysis revealed the formation of layered hydrates within the pores (Figure 11a,b). Their Mg/Al ratio of 2.30 closely matched that of hydrotalcite—a finding further supported by the DTG results in Figure 10b. This confirms that the reactive MgO derived from amorphous phases in the FNS actively participated in forming hydration products. Although SEM observation indicated a high content of C–S–H in the FCS, its structure appeared loose, with a Ca/Si ratio of approximately 1.70.

#### 2.4.4. FNS Activated by NaOH

It is reported that the hydration products in NaOH-activated mixtures mainly include C–(A)–S–H, N–A–S–H, (N, C)–A–S–H gels, hydrotalcite, and calcite [22,48]. However, in this study, only crystalline N–A–S–H (hydroxysodalite: Na_4_Al_3_Si_3_O_12_(OH)) and CaCO_3_ were detected in the XRD patterns (Figure 12).

In addition, hydrotalcite was not observed, which differs from the case in Ca-based activator systems. Notably, hydrotalcite-like phases have been reported in NaOH-activated blast furnace ferronickel slag (FNS) in the literature [50]. A comparison among NaOH-activated FNS, Ca-based activator-activated FNS, and NaOH-activated blast furnace FNS indicates that the CaO content in the alkali-activated binder plays a decisive role in the formation of hydrotalcite-like phases. Furthermore, no CH was detected in FNS pastes activated by Na(K)-based activators; therefore, the mass loss above 390 °C in the TG-DTG curves is mainly attributed to the decomposition of CaCO_3_. As shown in Figure 12b, the CaCO_3_ content was also relatively high in the hydration products of this system.

Compared to CH and CaO, NaOH—as a strong alkali—demonstrates a greater capacity to depolymerize the amorphous phase structure in FNS and cleave its Si–O and Al–O bonds. Simultaneously, Na ions enhance the reactivity of the amorphous phase, further facilitating its depolymerization. Given these effects, NaOH is frequently employed as one of the principal activators in alkali-activated materials. As illustrated in Figure 12b and Table 4, the non-evaporable water content in NaOH-activated FNS is also higher than that observed with Ca-based activators. However, NaOH is relatively expensive, costing approximately 5–6 times more than CH and CaO [20].

Based on SEM observations (Figure 13) and corresponding EDS analyses, two types of hydration products were identified. One is the reticulated C–S–H gel with a high sodium content—designated as (N, C)–(A)–S–H gel—which exhibits a Na/Si ratio of 0.56 (Figure 13b). It is also worth noting that the EDS results for area 5 in Figure 13a show very high contents of Mg. Therefore, this region may also contain a sodium-magnesium aluminosilicate gel (N–M–A–S), which is consistent with previous literature [51].

The other is crystalline N–A–S–H, characterized by high Na/Si (1.47) and Na/Al (1.53) ratios (Figure 13e). These elemental proportions are similar to those found in hydroxysodalite. Xu et al. [52] described the morphology of hydroxysodalite as resembling a “ball of yarn,” which is consistent with the observations in this study. This confirms that the crystalline N–A–S–H phase can be identified as hydroxysodalite. The significant formation of this crystalline phase is likely attributable to the elevated curing temperature.

#### 2.4.5. FNS Activated by Na_2_CO_3_

In the Na_2_CO_3_-activated system, the newly formed crystalline hydrates were identified as CaCO_3_ and crystalline N–A–S–H phases (gobbinsite, Na_3_Al_3_Si_5_O_16_·6H_2_O, and hydrosodalite, Na_4_Al_3_Si_3_O_12_(OH)), as shown in Figure 14a. Diffraction peaks corresponding to various sodium carbonate hydrates (Na_2_CO_3_·H_2_O, Na_2_CO_3_·7H_2_O, Na_2_CO_3_·10H_2_O and Na_3_H(CO_3_)_2_·2H_2_O) were also detected, indicating the presence of unreacted Na_2_CO_3_. The formation of these sodium carbonate hydrates consumes significant free water and releases considerable heat [53], leading to a shortened setting time of the FNC paste [54]. Notably, gaylussite (Na_2_Ca(CO_3_)_2_·5H_2_O)—which has been reported in Na_2_CO_3_-activated GBFS systems—was not observed here. This absence is likely due to insufficient Ca^2+^ availability in FNS [55].

Although more non-evaporable water was found in Na_2_CO_3_-activated FNS, it primarily originated from the dehydration of sodium carbonate hydrates, with only a minor portion attributable to the formation of C–S–H and N–A–S–H. Therefore, this non-evaporable water content has little correlation with the mechanical properties of the paste. As shown in Figure 15, both the size and number of C–S–H gels formed near the FNS particle surfaces were limited. Simultaneously, the paste structure was relatively loose, and no distinct connections between phases were observed in the matrix, indicating that its strength would be relatively low.

#### 2.4.6. FNS Activated by KOH

For FK, Figure 16a shows the presence of only crystalline K–A–S–H phases—specifically illite (KAl_2_(Si_3_Al)O_10_(OH)_2_) and illite-montmorillonite (KAl_4_(Si, Al)_8_O_10_(OH)_4_·4H_2_O)—along with CaCO_3_. Similar to FN, no hydrotalcite was detected in FK. However, as shown in Figure 16b, FK exhibited a lower non-evaporable water content than FN.

SEM-EDS observations of FK (Figure 17) revealed two distinct types of hydration products. The first is honeycomb-like C–S–H gel with a high K content (i.e., (K,C) –(A)–S–H gel), exhibiting a K/Si ratio of 0.29 (Figure 17c). It is worth noting that, based on the EDS results for area 7 in Figure 17b, the Mg content is very high. Therefore, this region may also contain a potassium-sodium-magnesium aluminosilicate gel (K–M–A–S), which is similar to the phenomenon observed in the FN system. Compared to FN, the C–S–H microstructure in FK is more compact, which is undoubtedly related to the incorporation of K^+^ ions.

The second product is rope knot-shaped K–A–S–H with high K/Si (0.59) and K/Mg ratios (Figure 17f). Its K/Si ratio is similar to that of illite. Götz et al. reported that illite exhibits a fibrous bundle morphology [56], which is generally consistent with the present observation. However, significant Mg was detected in its composition (Figure 17f), and a distinct knot appears in the middle of the fiber bundle (Figure 17e). Therefore, it can be inferred that this phase is likely an Mg-rich variety of illite. Although K-A-S-H gel typically exhibits a semi-crystalline or amorphous structure, the high-temperature environment provided by steam curing facilitates the further growth, rearrangement, and densification of gel particles, leading to a more ordered internal structure—i.e., a higher degree of crystallinity [57]. As a typical representative of crystalline K-A-S-H, illite predominantly appears as fiber bundles. In the present system, however, the high Mg content of the FNS promotes the formation of a Mg-rich K-A-S-H phase, which accounts for its distinctive knotted-fiber-bundle-like morphology.

### 2.5. Mechanical Property

**Figure 18:**
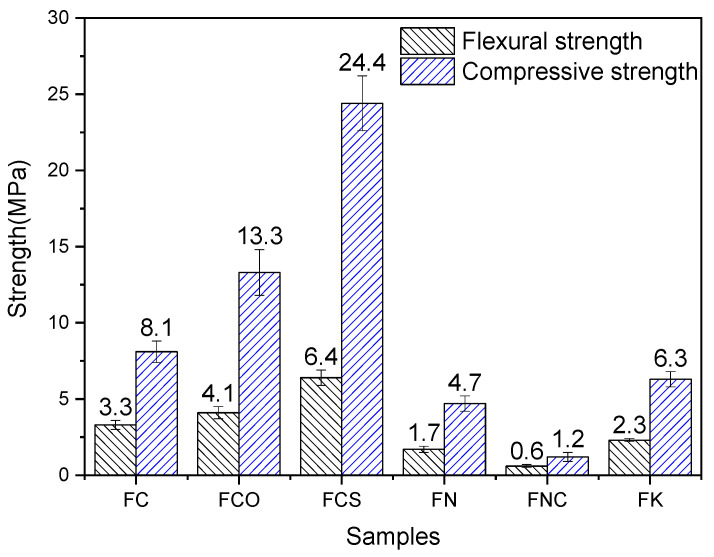
Strengths of alkali-activated FNS.

The compressive and flexural strengths of FNS activated by various alkaline activators under steam curing are presented in Figure 18. Due to the low reactivity of FNS, all samples exhibited low strength. Notably, FNS activated by Ca-based activators demonstrated higher mechanical strength but lower non-evaporable water content compared to those activated by Na- or K-based activators. This disparity is mainly due to the differences in the hydration products formed. It has been reported that C–S–H gels generally exhibit greater strength than N–A–S–H gels [58].

The compressive strength of Ca-based alkali-activated FNS pastes varied with the activator type. Among them, the paste co-activated by gypsum and Ca(OH)_2_ exhibited the highest strength, followed by that activated solely by CaO, while the paste activated solely by Ca(OH)_2_ showed the lowest strength. As indicated by the foregoing analysis, the combined activation by gypsum and Ca(OH)_2_ promoted the formation of a larger quantity of hydration products compared to either CaO or Ca(OH)_2_ alone. Furthermore, the higher strength observed in CaO-activated systems relative to Ca(OH)_2_-activated ones is also attributed to the greater amount of hydrates formed.

Among FNS pastes activated by Na(K)-based activators, FK exhibited higher strength than FN, even though its non-evaporable water content was lower—a finding consistent with results reported by Omur et al. [22,59]. The primary reasons for this strength difference are as follows: (1) The C–S–H structure in FN (Figure 13a) is relatively loose, whereas that in FK (Figure 17a,b) is more compact. (2) The FN matrix contains more pores (Figure 13c, 17.1% obtained from data analyzed using ImageJ 1.54p software (64-bit Windows version)) than FK (Figure 17d, 10.7%). (3) The N–A–S–H and K–A–S–H structures differ significantly: K–A–S–H exhibits higher mechanical performance. Compared with N–A–S–H, K–A–S–H possesses a higher skeletal density, stronger interatomic forces (i.e., stronger bonding), and a finer nanoparticle structure [60].

As shown in Figure 18, FNC exhibited very low strength. This is consistent with the scarce hydration products and the relatively loose microstructure observed in Figure 15.

Notably, the flexural strength of alkali-activated FNS followed a trend similar to its compressive strength. Samples prepared with Ca-containing activators exhibited higher flexural strength, with the FCS system showing the highest value. This can be attributed both to the greater amount of hydration products formed and to the formation of ettringite in FCS (Figure 10). The formation of ettringite in the FCS system contributes to the improved flexural strength of the alkali-activated FNS, which can be attributed to two primary mechanisms. First, the fibrous or rod-like ettringite crystals can bridge microcracks, hindering their further propagation and generating a fiber-reinforcement-like effect within the matrix. Second, the formation of ettringite is accompanied by volume expansion, particularly during the early stages of hydration. This expansion can, to some extent, offset the drying shrinkage of the alkali-activated FNS, thereby reducing the formation of shrinkage-induced microcracks [61].

In contrast, among systems activated by Na(K)-based activators, the FK system demonstrated relatively high flexural strength. While FK produced fewer hydration products than FN, its superior strength is likely linked not only to the type of hydration products but also to the unique “knotted fiber bundle” morphology observed in FK (Figure 17).

These knotted fiber bundles are embedded in the C–S–H gel matrix. During crack propagation, they must overcome the frictional stress at the bundle-matrix interface to be pulled out. This process dissipates more energy than the pull-out of ordinary fibers, functioning similarly to the anchoring mechanism of hooked steel fibers. Consequently, it is expected to significantly enhance the fracture toughness of concrete [62].

### 2.6. Discussion

#### 2.6.1. FNS Activated by Ca-Based Activators

The type of activator used is crucial in determining the phase composition and strength development of alkali-activated FNS. Recently, Ca-based activators have gained increasing attention for producing more sustainable cementitious materials, as they are typically less expensive and have lower alkalinity than their Na- or K-based counterparts [63]. However, binders activated solely by Ca-based materials often develop strength slowly. Notably, in this study, Ca-activated binders achieved higher ultimate strength than those activated by Na or K (Figure 18). Beyond the effect of steam curing, this is primarily attributed to the nature of the hydration products: the Ca-activated system is a “high-calcium” system that forms high-strength C–S–H as the main hydrate, whereas the Na(K)-activated system is a “low-calcium” system that yields the lower-strength N–A–S–H or K–A–S–H gel [58,64]. Consequently, the CaO content in the activator is a key factor controlling the final strength of the alkali-activated FNS paste.

In addition, hydrotalcite and AFm were detected in the high-calcium system but not in the low-calcium system. This is primarily attributed to the competitive reactions arising from the limited Al_2_O_3_ content in the system. Since the formation of both hydrotalcite and AFm phases requires the participation of aluminum, and the Al_2_O_3_ content in ferronickel slag is quite limited, these aluminum-containing phases must compete with other hydration products for aluminum sources [65]. In low-calcium systems, the primary hydration product is N(K)-A-S-H gel, the formation of which consumes most of the available aluminum, thereby inhibiting the generation of hydrotalcite and AFm. In contrast, in calcium-rich systems, the main hydration product is C–S–H gel, which has a lower demand for aluminum [66]. Consequently, the formation of hydrotalcite and AFm phases faces relatively less competition in such systems. However, it should be noted that although C–S–H can incorporate Al to form C–A–S–H in “high-calcium” systems, the formation of hydrotalcite also competes with C–S–H for Al. This reduces the amount of Al available for C–S–H in the system, thereby leading to the formation of low-Al C–A–S–H [67]. However, due to factors such as the significant difference in ionic radius between Ca^2+^ and Mg^2+^, Ca^2+^ is generally unable to substitute for Mg^2+^ in the formation of hydrotalcite [68,69].

#### 2.6.2. FNS Activated by Na(K)-Based Activators

(1) NaOH

Both NaOH and KOH are strong bases. However, for the same mass dosage, NaOH has a higher molar concentration, resulting in the formation of more hydration products in FN, as shown in Figure 12 and Figure 16. Although FNS activated by Na_2_CO_3_ also contains considerable non-evaporable water, its hydration products are rich in hydrated carbonate phases. While these phases contribute little to strength development, they can accelerate paste hardening.

NaOH is one of the most widely used alkaline activators. Its high-pH solution promotes rapid reaction, giving alkali-activated slag paste relatively high early-age strength. However, studies indicate that NaOH-activated GBFS often exhibits lower later-age strength, which is attributed to its coarser pore structure. The strength of alkali-activated slag is influenced by factors such as the type, dosage, and addition method of the activator. A high dosage creates a highly alkaline environment that accelerates the dissolution of the amorphous phase and promotes the polymerization of hydration products. However, excessive alkali tends to favor the formation of high-alkalinity, low-strength polymerization products (e.g., N–A–S–H, pH > 14) over low-alkalinity, high-strength products (e.g., C–S–H, pH ≈ 13 [48]). Moreover, it can lead to efflorescence due to free alkali remaining in the system.

(2) Na_2_CO_3_

Na_2_CO_3_ is less corrosive than NaOH or KOH, offering a safer working environment. Although previous studies indicate that Na_2_CO_3_-activated GBFS can achieve relatively high strength and durability, it suffers from significantly prolonged setting and slower strength development [55]. Due to these slow reaction kinetics, it is generally not considered a practical primary activator in alkali-activated systems, especially with FNS as the precursor. Interestingly, in this study, even 80 °C steam curing did not improve the strength development of Na_2_CO_3_-activated FNS, with compressive strength remaining near 1 MPa (Figure 18)—a result consistent with previous reports [70]. Notably, despite its lower pH than NaOH, the carbonate ions (CO_3_^2−^) in Na_2_CO_3_ can promote the formation of carbonation products (e.g., C_3_A·CaCO_3_·12H_2_O). This carbonation effect may explain the higher long-term strength sometimes observed in Na_2_CO_3_-activated GBFS compared to NaOH-activated systems [71].

(3) KOH

Although KOH has higher alkalinity than NaOH [72], using the same mass results in a lower molar concentration for KOH than for NaOH. Consequently, the KOH-activated FNS (referred to as FK) exhibited a reduced quantity of hydration products (Figure 12 and Figure 16) and an extended setting time. In contrast, NaOH is more effective in breaking the Ca–O, Si–O, and Al–O bonds in FNS, which releases more heat and accelerates hydrate formation at early ages (Table 4).

Moreover, the lower charge density of K^+^ compared to Na^+^ weakens ion-dipole interactions and reduces solution viscosity [73]. Furthermore, the higher zeta potential in KOH-activated slag paste promotes the adsorption of K^+^, leading to reduced van der Waals forces and enhanced electrostatic repulsion between particles [74]. Consequently, KOH-activated pastes generally exhibit lower yield stress and higher fluidity. In the present study, however, the fluidity of all mixes was high, which resulted in only a marginal difference between the FK and FN systems.

Notably, despite producing fewer hydration products, FK exhibited higher strength than FN. Omur et al. reported a minimal difference in porosity between KOH- and NaOH-activated pastes under similar conditions [22], suggesting that the intrinsic properties of the strength-contributing hydration products—such as C–S–H, N–A–S–H, and K–A–S–H—play a decisive role in mechanical performance. In particular, the C–S–H gels in KOH-activated pastes exhibit a denser microstructure. Moreover, compared to N–A–S–H, the K–A–S–H phase demonstrates superior mechanical properties. The underlying mechanisms primarily include K-A-S-H displaying a finer nanogranular structure than N–A–S–H (3 nm vs. 30 nm particle diameters; 5 nm vs. 50 nm average pore diameters), higher skeletal density (2.3 vs. 2.02 g/cm^3^), and higher failure stress (19.5 GPa vs. 10 GPa) and failure strain (0.28 vs. 0.22) [49,60,75,76].

In addition, the knotted-fiber-bundle-like K–A–S–H formed in the FK system offers potential advantages for enhancing the crack resistance of concrete, as evidenced by the following:

(i) Fibrous ettringite is generally recognized for improving the flexural strength of concrete, and the crystalline K–A–S–H exhibits a superior knotted fiber bundle structure similar to that of hooked steel fibers.
(ii) Despite the relatively low amount of hydration products formed in the FK system, its flexural strength remains significantly higher than that of the FN system.

#### 2.6.3. Limitations of This Study and Future Research

(1) Given the low CaO and Al_2_O_3_ contents in FNS, it lacks sufficient calcium and aluminum sources required for the formation of the primary hydration products of alkali-activated materials, such as C–S–H and N–A–S–H gels. Therefore, FNS is not suitable for use as the sole precursor in alkali-activated systems. For future applications, it is recommended that FNS be combined with aluminum-rich precursors (e.g., fly ash or metakaolin) or calcium-rich precursors (e.g., GBFS or steel slag) to produce alkali-activated materials with improved performance.

(2) Although using a fixed mass dosage of activator is more common in practical applications, this approach may result in variations in the effective alkali concentration among different activators. Therefore, future studies are encouraged to investigate activator dosages based on equivalent molar concentrations to better elucidate the influence of activator type on the hydration process and macroscopic properties of alkali-activated materials.

(3) Alkali-activated FNS, especially when activated by calcium hydroxide or calcium oxide, is highly prone to carbonation. To minimize its effects, it is recommended that: First, samples should be stored in a CO_2_-free environment. Second, their exposure to carbon dioxide during preparation and transfer must be minimized.

(4) In this study, Ca-based activators have demonstrated significant potential in activating FNS. Therefore, it is essential to further investigate the influence of such activators on the properties of FNS under standard curing conditions, including the evolution of hydration products over time, the development of microstructure, and the correlation between microstructure and macroscopic properties, with particular attention to long-term durability.

Furthermore, experimental results demonstrate that the combination of CH and CaSO_4_·2H_2_O achieves the highest mechanical strength in FNS activation. However, since all reagents used in this study were of chemical grade, their high cost is a major limiting factor for large-scale application. To improve economic feasibility, future work could focus on exploring an all-solid-waste system comprising carbide slag (an industrial by-product rich in CH), desulfurization gypsum or phosphogypsum, and FNS.

(5) After 7 days of steam curing at 80 °C, the knotted fibrous bundles of K–A–S–H formed in KOH-activated FNS exhibit a reinforcing effect similar to that of hooked steel fibers in cementitious matrices. To further validate the potential of the knotted-fiber-bundle-like K–A–S–H phase in enhancing crack resistance, future research could explore the following directions. First, knotted-fiber-bundle-like K–A–S–H could be synthesized using KOH as the activator, metakaolin (rich in reactive SiO_2_ and Al_2_O_3_) as the precursor, and industrial-grade light-burned MgO as an additive, under the curing conditions adopted in this study (80 °C steam curing for 7 days). Its micromechanical properties should then be characterized. Second, the synthesized K–A–S–H could be incorporated as an additive into cement-based materials to evaluate its effect on the crack resistance of concrete. Finally, using metakaolin as a supplementary cementitious material (SCM) in combination with the same activator system (KOH and light-burned MgO) and curing conditions, future studies should investigate whether the knotted K–A–S–H phase can form in situ within cement-based materials and further assess its contribution to improving concrete crack resistance.

## 3. Conclusions

This study aims to investigate the existence forms of MgO in ferronickel slag (FNS) and their effects on hydration, as well as to evaluate the potential of various activators to activate FNS. First, the composition of FNS with varying particle sizes was characterized. Subsequently, six activators (Ca(OH)_2_, CaO, NaOH, Na_2_CO_3_, KOH, and Ca(OH)_2_ + gypsum) were used to activate FNS under 80 °C steam curing for 7 days. The hydration products, microstructure, and mechanical properties of the resulting alkali-activated materials were then analyzed. The main conclusions are as follows:

(1) Analysis of the phase composition and chemical composition of FNS with different particle sizes confirmed the presence of amorphous MgO, which can participate in the formation of hydrotalcite-like minerals.

(2) Regarding setting time, the values for FNS activated by CaO, NaOH, Ca(OH)_2_ + gypsum, KOH, Ca(OH)_2_, and Na_2_CO_3_ increased progressively under room temperature conditions. Specifically, the final setting time was 2.5 h for the CaO-activated system and 18.5 h for the NaOH-activated system, whereas it exceeded 24 h for all other activators. In terms of fluidity, mixtures activated by Na(K)-based activators exhibited significantly higher values than those activated by Ca-based ones. Among them, the mixtures activated by CaO and Ca(OH)_2_ + gypsum showed the lowest fluidity, both at 120 mm.

(3) Under steam curing at 80 °C for 7 days, the activation efficiency of different activators on FNS varied significantly. The highest mechanical strength was achieved by the system co-activated with Ca(OH)_2_ and gypsum, which was more effective than using CaO or Ca(OH)_2_ alone. In contrast, alkaline activators such as KOH and NaOH led to progressively lower mechanical performance.

(4) Hydrotalcite and AFm phases were only detected in FNS activated by Ca-based activators (Ca(OH)_2_, CaO, and Ca(OH)_2_ + gypsum), not in those activated by Na(K)-based activators (NaOH, Na_2_CO_3_, and KOH).

(5) Interestingly, in NaOH-activated FNS, crystalline N–A–S–H with a morphology resembling a “ball of yarn” was observed, while in KOH-activated FNS, K–A–S–H formed in the shape of “knotted fiber bundles.” Compared to simple fiber bundles, such knotted structures may more effectively enhance crack resistance in cementitious materials.

## 4. Materials and Methods

### 4.1. Materials

The ferronickel slag (FNS) used in this study was water-quenched electric arc furnace (EAF) slag with a density of approximately 2.9 g/cm^3^, supplied by Jiangsu Rongda New Material Co., Ltd. (Nantong, China).

To assist in identifying the phase composition of FNS, this study selected FNS sand of three particle size fractions—coarse (>4.75 mm), medium (0.15–4.75 mm), and fine (<0.15 mm)—for comparative analysis.

The FNS powder used in the alkali activation experiments originated from the same factory and followed the same production process as the aforementioned FNS sand. It should be clearly noted that this FNS powder was not obtained by separately grinding the FNS sand based on particle size; instead, it was produced by mixing FNS of all particle sizes and grinding them together.

Deionized water and analytical reagent-grade compounds—Ca(OH)_2_ (CH, dry powder, purity > 95%), CaO (dry powder, purity > 98%), NaOH (dry powder, purity > 96%), KOH (dry powder, purity > 90%), Na_2_CO_3_ (dry powder, purity > 98%), and CaSO_4_·2H_2_O (dry powder, purity > 99%)—were each employed as an alkaline activator.

Alkali-activated mortar was prepared using ISO standard sand to test its fluidity. The standard sand had a fineness modulus of 2.75, an SiO_2_ content of 97%, a loss on ignition of 0.10%, and a clay content of 0.10%.

### 4.2. Sample Preparation and Test Methods

#### 4.2.1. Sample Preparation

Both FNS and GBFS are solid wastes rich in MgO, and previous studies have shown that under steam curing at 80 °C for 7 days, the reactivity of MgO in GBFS can be effectively activated to participate in hydration reactions [12,14]. Inspired by this, the same curing conditions (steam curing at 80 °C for 7 days) were adopted in this study, with the aim of activating the MgO in FNS and investigating its role in the alkali-activated system. It should be emphasized that no experiments related to GBFS were conducted in this study; GBFS is mentioned solely to explain the rationale behind the chosen curing conditions.

**Figure 19:**
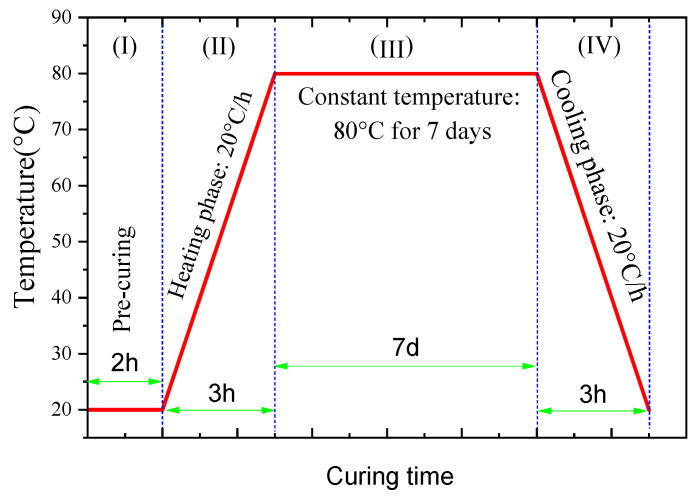
Schematic diagram for steam curing.

Steam curing is typically carried out within a temperature range of 45–80 °C and can be regarded as a curing method that achieves high-temperature, high-humidity conditions by maintaining a saturated water vapor environment [77]. In this study, the specific parameters of the steam curing regime at 80 °C for 7 days are shown in Figure 19. The entire steam curing process is divided into four stages: the pre-curing period (I), heating period (II), constant temperature curing period (III), and cooling period (IV). The specific parameters are set as follows:

Pre-curing period (I): Immediately after casting, the alkali-activated material (AAM) specimens were transferred to a constant temperature and humidity curing chamber (ZKY-400, Cangzhou Yixuan Testing Instrument, Cangzhou, China). The pre-curing conditions were a temperature of 20 ± 2 °C and a relative humidity of >95%, with a pre-curing time of 2 h.

Heating period (II): After the pre-curing period, the AAM specimens were transferred to a concrete rapid curing chamber (HJ-84, Cangzhou Dongyi Rongke Experimental Instrument, Cangzhou, China) and placed on a shelf above the water surface. The temperature and humidity inside the chamber were regulated by controlling the temperature of the water. During the heating period, the temperature was increased at a rate of 20 °C/h, taking approximately 3 h to reach the target temperature of 80 °C.

Constant temperature curing period (III): The curing conditions were a constant temperature of 80 °C and a relative humidity of >95%, with a constant temperature curing time of 7 days.

Cooling period (IV): The temperature was decreased at a rate of 20 °C/h, taking approximately 3 h to cool to room temperature.

Furthermore, FNS is considered an environmentally friendly alternative to ordinary Portland cement (OPC). Notably, key ionic species in cement that influence FNS hydration—such as Na^+^, K^+^, Ca^2+^, and SO_4_^2−^—are also common activators for alkali-activated materials (AAMs) based on FNS. Given this shared chemistry, this study selected CH, CaO, NaOH, KOH, Na_2_CO_3_, and a CH-gypsum composite as alkaline activators to investigate both the reaction mechanism of FNS in concrete and the activation efficacy of various agents on FNS. Additionally, C–S–H gel is the primary phase contributing to the strength of alkali-activated materials. CaO not only serves as a key component of C–S–H gel but is also the main constituent of calcium-based alkaline activators. Therefore, given the inherently low CaO content of FNS, ensuring adequate strength development in the alkali-activated FNS system is a critical consideration. This also forms the primary rationale for selecting CaO or CH as the activator in this study.

For alkaline-activated GBFS, the dosage of CaO or CH—typically regarded as potential activators—is generally limited to less than 10% of the total binder mass [78]. However, given that FNS exhibits substantially lower reactivity than GBFS, a dosage of 10% was specified for both CaO and CH in this investigation. For consistency in comparison, the dosage of all other activators was also set at 10%. Meanwhile, a 5% gypsum dosage is widely adopted in the production of conventional OPC and alkali-activated slag cement [79]; accordingly, a blend of 5% gypsum and 10% CH was employed to investigate the synergistic effects of sulfate and lime on FNS.

Considering the long setting time of alkali-activated FNS paste, sand-free paste prism specimens with dimensions of 40 mm × 40 mm × 160 mm were prepared for mechanical property tests at a fixed water-binder ratio of 0.3 using the six activators listed in Table 6, in accordance with the Chinese national standard GB 17671-2021 [80]. It is noteworthy that NaOH, Na_2_CO_3_, and KOH were dissolved in water and cooled to room temperature prior to mixing, whereas Ca(OH)_2_, CaO, and gypsum were directly blended with FNS powder during sample preparation.

#### 4.2.2. Test Methods

The setting time of all paste mixtures was determined via a Vicat apparatus according to Chinese standard GB/T 1346-2024 [81].

The fluidity of alkali-activated FNS paste was determined via the mortar fluidity method, which was performed using the jump table test in accordance with the Chinese national standard GB/T 2419-2024 [82]. To ensure that all AAM possess adequate workability for construction, the mix proportion used in this study for testing the fluidity of AAM mortar was 450 g of the mixture of FNS and alkali activator, 1350 g of standard sand, and 225 g of water.

After 7 days of steam curing, the mechanical properties of alkali-activated FNS paste with the dimension of 40 mm × 40 mm × 160 mm were performed after demolding according to Chinese standard GB/T 17671-2021 [80].

The paste samples, after being subjected to steam curing for 7 days, were treated by solvent exchange with absolute ethanol to halt hydration. They were then crushed, and the resulting specimens were used for XRD, SEM-EDS, and TG-DTG analysis.

It should be noted that all procedures, including the preparation of AAM specimens, as well as subsequent tests for setting time, fluidity of mortar, mechanical properties, and the crushing and grinding of specimens for microstructural analysis, were conducted under controlled environmental conditions of 20 ± 2 °C and 55–65% RH.

The phase composition of FNS and its alkali-activated hydration products was characterized by X-ray diffraction (XRD) using a D8-Discover diffractometer with a scanning speed of 3°/min. The relative content of each phase in FNS was determined via the XRD/Rietveld method, employing 10 wt% corundum as an internal standard.

The morphology of FNS and its alkali-activated hydration products and related phase compositions were checked by a Sirion (FEI Company, Hillsboro, OR, USA) field emission scanning electron microscope (SEM) and energy dispersive X-ray spectra (EDS) (FEI Company, Hillsboro, OR, USA).

The thermogravimetric/derivative thermogravimetric (TG/DTG) data were carried out by heating the samples from 60 °C (FNS from 105 °C) to 1000 °C with a constant rate of 10 °C/min using STA 449 F3 Jupiter^®^ (NETZSCH-Gerätebau GmbH, Selb, Germany).

The porosity of the AAM paste, defined as pores larger than 20 pixels in the SEM images, was statistically analyzed using Fuji ImageJ (64-bit Windows version).

## Figures and Tables

**Table 1:** The chemical compositions of FNS powder, wt.%.

Materials	CaO	SiO_2_	Al_2_O_3_	SO_3_	Fe_2_O_3_	MgO	Na_2_O	K_2_O	Cr
FNS	11.49	47.61	6.56	0.52	13.24	15.94	0.80	0.18	0.70

**Table 2:** Chemical composition of FNS particles with various sizes, wt.%.

Type of FNS	SiO_2_	MgO	Fe_2_O_3_	Al_2_O_3_	CaO	P_2_O_5_	K_2_O	TiO_2_	MnO	SO_3_	Cr
<0.15 mm	53.95	17.88	9.41	8.82	6.53	1.02	0.44	0.28	0.63	0.32	0.59
0.15~4.75 mm	50.42	20.72	8.48	8.09	5.98	1.07	0.29	0.23	0.60	0.20	0.52
>4.75 mm	53.22	19.72	9.69	8.26	5.99	1.09	0.25	0.23	0.66	0.26	0.61

**Table 3:** Setting time and fluidity of alkali-activated FNS paste.

Samples	Setting Time (h)		Mortar Fluidity (mm)
	Initial	Final	
FC	35.0	46.0	150
FCO	1.60	2.50	120
FCS	32.0	42.0	120
FN	12.0	18.5	300
FNC	48.0	59.0	300
FK	33.0	45.0	300

**Table 4:** Mass loss at a specific temperature range in various alkali-activated FNS pastes, wt.%.

**Samples**	**Mass Loss**	**<390 °C**	**390~490 °C**	**>490 °C**
FC	6.60	2.80	1.00	2.80
FCO	7.40	3.30	1.30	2.80
FCS	9.90	4.90	0.90	4.10
**Samples**	**Mass Loss**	**<390 °C**	**>390 °C**	
FN	9.00	6.00	3.00	
FK	10.3	5.80	4.50	
FNC	7.80	4.50	3.30	

**Table 5:** The temperature ranges for the phase and the related reactions.

Temperature Ranges	Phases and Reactions
27~92 °C	Water loss from Na_2_CO_3_·10H_2_O, Na_2_CO_3_·7H_2_O, Na_2_CO_3_·10H_2_O [28]
50~220 °C	The loss of bound water from C–S–H [29], N–A–S–H [30] and K–A–S–H [31]
50~150 °C	Decomposition of ettringite [29]
50~220 °C	Decomposition of gypsum [32]
120~230 °C	Thermal decomposition of NaHCO_3_ [33,34]
160~220 °C	Water loss of carboaluminate hydrates (monocarbonate and hemicarbonate) [35]
200~600 °C	Decomposition of Na_3_H(CO_3_)_2_·2H_2_O [36]
285~380 °C	Decomposition of hydrotalcite [37]
380~490 °C	Dehydroxylation of CH [38]
700~900 °C	Decarbonation of carbonates [38]

**Table 6:** Mix proportions (%) of various alkali-activated FNS.

Sample	FNS	CH	CaO	NaOH	KOH	Na_2_CO_3_	Gypsum	Water
FC	90	10	0	0	0	0	0	30
FCO	90	0	10	0	0	0	0	30
FCS	85	10	0	0	0	0	5	30
FN	90	0	0	10	0	0	0	30
FNC	90	0	0	0	0	10	0	30
FK	90	0	0	0	10	0	0	30

## Data Availability

All data generated or analyzed during this study are included in this submitted article.

## Abbreviations

The following abbreviations are used in this manuscript:

FNS	Ferronickel slag
EAF	Electric arc furnace
SCM	Supplementary cementitious material
GBFS	Ground granulated blast-furnace slag
OPC	Ordinary Portland cement
ASTM	American Society for Testing and Materials
AAMs	Alkali-activated materials
C–S–H	Calcium silicate hydrate
N–A–S–H	Sodium aluminosilicate hydrate
K–A–S–H	Potassium aluminosilicate hydrate
CH	Calcium hydroxide
AFt	Ettringite
AFm	Monosulfoaluminate hydrate
XRF	X-ray fluorescence
LOI	Loss on ignition
XRD	X-ray diffractometer
SEM-EDS	Scanning electron microscope coupled with an X-ray energy dispersive spectrometer
TG-DTG	Thermogravimetric and derivative thermogravimetric analyses
